# Re: ASGR1 Deficiency Promotes Liver Fibrosis by Enhancing Pro‐Inflammatory Arachidonic Acid Metabolism and ALP Secretion

**DOI:** 10.1002/iid3.70543

**Published:** 2026-09-29

**Authors:** Urwa Ihsan, Ayesha Asif

**Affiliations:** ^1^ Department of Medicine Allama Iqbal Medical College Lahore Pakistan

## Conflicts of Interest

The authors declare no conflicts of interest.

To the Editor,

1

We read with great interest Zhu and colleagues' article, “ASGR1 Deficiency Promotes Liver Fibrosis by Enhancing Pro‐Inflammatory Arachidonic Acid Metabolism and ALP Secretion” [1], which proposes an “ASGR1–arachidonic acid–ALP” axis underlying liver injury from ASGR1‐targeted therapies, with serum ALP suggested as an early safety biomarker. This is a valuable hypothesis for a field actively developing ASGR1 inhibitors. However, given that the authors explicitly cite adherence to the ARRIVE 2.0 guidelines [2], several methodological omissions in the animal studies must be addressed before this framework can be considered biomarker‐ready.

First, the manuscript reports a power calculation specifying six mice per group, with six actually used “to account for potential attrition” [1], yet no exclusion criteria or attrition numbers are reported anywhere in the study. This is not a minor omission: Holman and colleagues showed in a simulation and meta‐analysis of preclinical research that even small, nonrandom animal losses can substantially distort effect sizes and inflate false positive results [3]. The concern is visible in the paper's own data: the BDL cohort (Figure 4) reports *n* = 4 (WT), *n* ≥ 6 (Asgr1^+/−^), and *n* ≥ 3 (Asgr1^−/−^)—falling below the prespecified minimum of six per group, without any explanation. Notably, the smallest group is the Asgr1^−/−^ genotype driving the paper's central finding, while ARRIVE 2.0 requires authors to disclose and justify departures from planned sample sizes [2].

Second, while random allocation via a number table is described, there is no statement on whether this sequence was concealed from those performing injury induction. Concealment and blinding protect against different biases, and systematic reviews of preclinical research consistently find that studies omitting allocation concealment report larger, less reliable effect sizes than those reporting it [4].

Third, as all in vivo experiments were performed exclusively on male C57BL/6 mice. As a well‐documented sexual dimorphism occurs in hepatic metabolism and fibrogenesis in mice and humans, the translational relevance of these findings to female subjects remains to be determined [5].

None of this undermines the plausibility of the underlying biology. But given the authors' explicit goal of proposing ALP for clinical drug‐safety monitoring, we would encourage reporting per‐group attrition, clarifying the BDL sample‐size discrepancy, and specifying allocation concealment—steps that would meaningfully strengthen confidence in a mechanism that may prove clinically important.

## Author Contributions

**Urwa Ihsan:** writing – review and editing, writing – original draft and conceptualization. **Ayesha Asif:** writing – review and editing, writing – draft and conceptualization. Both authors have read and approved the final manuscript.

## Funding

The authors have nothing to report.

## Ethics Statement

Ethical approval was not required for this manuscript, as it involves no new human or animal experimentation.

## Consent

Informed consent was not required for this manuscript, as it involves no new human or animal experimentation.

## Data Availability

Data sharing is not applicable to this article as no new data sets were generated or analyzed during the current study.
